# IL-12 Expressing oncolytic herpes simplex virus promotes anti-tumor activity and immunologic control of metastatic ovarian cancer in mice

**DOI:** 10.1186/s13048-016-0282-3

**Published:** 2016-10-27

**Authors:** Eric D. Thomas, Selene Meza-Perez, Kerri S Bevis, Troy D. Randall, G. Yancey Gillespie, Catherine Langford, Ronald D. Alvarez

**Affiliations:** 1 Department of Obstetrics and Gynecology, Division of Gynecologic Oncology, University of Alabama at Birmingham, 1700 6th Avenue South, Room 10250, Birmingham, AL 35233 USA; 2 Department of Medicine, Division of Clinical Immunology and Rheumatology, University of Alabama at Birmingham, Birmingham, USA; 3 Department of Neurosurgery, University of Alabama at Birmingham, Birmingham, USA

**Keywords:** Immunotherapy for treatment of ovarian cancer, Oncolytic herpes simplex virus

## Abstract

**Background:**

Despite advances in surgical aggressiveness and conventional chemotherapy, ovarian cancer remains the most lethal cause of gynecologic cancer mortality; consequently there is a need for new therapeutic agents and innovative treatment paradigms for the treatment of ovarian cancer. Several studies have demonstrated that ovarian cancer is an immunogenic disease and immunotherapy represents a promising and novel approach that has not been completely evaluated in ovarian cancer. Our objective was to evaluate the anti-tumor activity of an oncolytic herpes simplex virus “armed” with murine interleukin-12 and its ability to elicit tumor-specific immune responses. We evaluated the ability of interleukin−12-expressing and control oncolytic herpes simplex virus to kill murine and human ovarian cancer cell lines in vitro. We also administered interleukin−12-expressing oncolytic herpes simplex virus to the peritoneal cavity of mice that had developed spontaneous, metastatic ovarian cancer and determined overall survival and tumor burden at 95 days. We used flow cytometry to quantify the tumor antigen-specific CD8^+^ T cell response in the omentum and peritoneal cavity.

**Results:**

All ovarian cancer cell lines demonstrated susceptibility to oncolytic herpes simplex virus in vitro. Compared to controls, mice treated with interleukin−12-expressing oncolytic herpes simplex virus demonstrated a more robust tumor antigen-specific CD8^+^ T-cell immune response in the omentum (471.6 cells vs 33.1 cells; *p* = 0.02) and peritoneal cavity (962.3 cells vs 179.5 cells; *p* = 0.05). Compared to controls, mice treated with interleukin−12-expressing oncolytic herpes simplex virus were more likely to control ovarian cancer metastases (81.2 % vs 18.2 %; *p* = 0.008) and had a significantly longer overall survival (*p* = 0.02). Finally, five of 6 mice treated with interleukin−12-expressing oHSV had no evidence of metastatic tumor when euthanized at 6 months, compared to two of 4 mice treated with sterile phosphate buffer solution.

**Conclusion:**

Our pilot study demonstrates that an interleukin−12-expressing oncolytic herpes simplex virus effectively kills both murine and human ovarian cancer cell lines and promotes tumor antigen-specific CD8^+^ T-cell responses in the peritoneal cavity and omentum, leading to reduced peritoneal metastasis and improved survival in a mouse model.

## Background

Ovarian cancer remains the most lethal cause of gynecologic cancer mortality with a median 5-year survival of 45 % for all stages. The American Cancer Society estimated that 21,290 women will be diagnosed with ovarian cancer in 2015 and that 14,180 will die of the disease [1]. Although advances in surgical resection and conventional chemotherapy have improved progression-free survival for the majority of women, most will eventually develop resistance to traditional cytotoxic chemotherapies and succumb to their disease [2]. Thus, there is a clear and urgent need for new therapeutic agents and innovative treatment paradigms for women with ovarian cancer.

Immunotherapy is a promising and novel approach not fully evaluated in the context of ovarian cancer. Ovarian cancer is an immunogenic disease as demonstrated by the expression of tumor-associated antigens such as mesothelin, CA-125, MUC-1, NY-ESO-1, and HER2/NEU, along with the discovery of antibodies and T-cells isolated from peripheral blood of ovarian cancer patients that react with these antigens [3]. Immunotherapy represents an innovative approach as a therapeutic modality [4, 5]. Several investigators have demonstrated that immune response in ovarian cancer correlates with patient outcomes. For example, the presence of tumor infiltrating lymphocytes (TILs), particularly CD8^+^ TILs, is associated with improved overall survival in women with advanced ovarian cancer [6–8]. Conversely, others have demonstrated that a lack of TILs correlates with worse outcomes and resistance to chemotherapy [9], suggesting that the cytotoxic effects of chemotherapy may be aided by CD8^+^ T-cells responding to released tumor- associated antigens [10]. Unfortunately, not all T-cells promote immunity, as the recruitment of immunosuppressive regulatory T-cells (Tregs), correlates with reduced survival in ovarian cancer [11]. Thus, there appears to be a clear rationale for evaluating virus-mediated immunotherapy in ovarian cancer. It is our contention that oncolytic HSV that expresses a potent cytokine, Interleukin-12, would accomplish three important intratumoral changes: i) direct tumor cell lysis by the virus, ii) generation of a tumor debris field and increased exposure of tumor-associated antigens to immune effector cells allowing for Matzinger “danger signal” immune activation, and iii) augmentation of T_H_1-type anti-tumor response to antigen and co-stimulation by intratumoral IL-12 expression (Signal 3) [12–14].

Importantly, immune responses to ovarian cancer must be targeted to the peritoneal cavity and the omentum, which are the primary sites of ovarian cancer metastasis [15]. However, the peritoneal cavity is a unique immunologic environment, with an abundance of interleukin (IL)-10-producing macrophages and B cells as well as Tregs [16, 17]. Ovarian carcinoma may take advantage of the unique properties of the omentum to suppress development of anti-tumor immunity [15]. Thus, novel treatments for ovarian carcinoma must be effective in the unique immunological environment of the peritoneal cavity and omentum.

Oncolytic viruses are designed to selectively replicate in tumor cells and induce cell death through apoptosis, autophagy, and/or necrosis. In addition to the cytotoxic effects achieved by oncolytic viruses, they also illicit an antitumor effect through the production of neoantigens and provide subsequent anti-tumor immune response [12, 13, 18]. These immunogenic effects can be enhanced when oncolytic viruses are transformed to express co-stimulatory cytokines, such as IL-12. Clinical trials have evaluated and confirmed the safety of oncolytic viruses, such as adenovirus, reovirus, and measles virus for the treatment of ovarian carcinoma [19, 20]. Genetically engineered oncolytic herpes simplex viruses (oHSV) have also demonstrated an ability to infect and kill several cancer cell types, including ovarian and melanoma [21–23]. Investigators have modified oHSV to incorporate various constructs in order to enhance their cytotoxic and immune-stimulating effects. For example, preclinical studies show that IL-12 expressing oHSV (M002) is effective in murine and primate models of brain tumors and enhances the influx of both CD4^+^ and CD8^+^ T-cells [12, 24]. Furthermore, the oHSV M002 has been shown to mediate superior anti-tumor effects when compared to other non-cytokine HSVs [13]. These findings, in addition to our prior preclinical studies of M002 in other tumor models, provided the rational for investigating the IL-12 oHSVs in ovarian cancer [12, 13].

Specifically, we tested in this pilot whether IL-12 expressing oHSV strains could infect and kill ovarian cancer cell lines in vitro and promote anti-tumor immunity in an ovarian cancer mouse model. Our results demonstrated that each evaluated ovarian cancer cell line exhibited sensitivity to oHSV when tested in vitro. Mice treated with intraperitoneal (IP) IL-12 expressing oHSV demonstrated a more robust tumor antigen-specific CD8^+^ T-cell immune response within all evaluated tissues. Finally, mice treated with IL-12 expressing oHSV were more likely to control ovarian cancer metastases and had a significantly longer overall survival.

## Methods

### Oncolytic herpes simplex viruses (oHSVs)

Wild-type herpes simplex virus (HSV) causes potentially life-threatening encephalitis, consequently attenuation is a prerequisite of oncolytic HSV development. To accommodate this effect, several mutations within the following viral genes within HSV have been described: thymidine kinase [25], ribonucleotide reductase [26], UTPase [27], or γ 34.5 gene [28, 29]. In our study, we utilized the genetically engineered oHSV strains, R3659 and G207, with deletions in the diploid γ34.5 gene (Δγ34.5 oHSV) [30]. The native γ34.5 gene exists in two copies and normally encodes a multifunctional protein of 263 amino acids that enable the HSV to efficiently replicate in infected cells even in the presence of a host antiviral response. In wild type HSV infected non-cancerous cells, protein kinase R is stimulated by the production of double stranded viral RNAs and phosphorylates eukaryotic initiation factor 2 alpha (eIF-2α) to block protein synthesis. Wild-type HSV uses the γ34.5 gene product (Infected Cell Protein 34.5) to bind Protein Phosphatase-1 and directing it to de-phosphorylate eIF-2α, thus reactivating eIF-2α.. Host cell protein shutoff is reversed allowing for viral replication to proceed. γ34.5-deleted HSVs are safe in normal cells since it cannot replicate efficiently; however, tumor cells inactivate PKR upstream generally by activating or mutating *ras* which makes them very susceptible to infection and unfettered replication by γ34.5-deleted HSVs.

In our experiments we also utilized ψ34.5 gene modified oHSVs engineered to express either murine IL-12 (M002), [12, 13] or human IL-12, (M032) as a means to augment the host immune responses to these viruses [24, 31]. Using an intracranial syngeneic neuroblastoma murine model, Parker et al. demonstrated improved survival in mice treated with M002, along with an influx of CD8^+^ T-cells [12].

### Ovarian cancer cell lines

We utilized several ovarian adenocarcinoma cell lines, both murine and human, along with ovarian cancer cells harvested from female MISIIR-TAg mice, a spontaneous ovarian cancer mouse model, to assess the in vitro cytotoxicity of oHSV. We specifically evaluated four syngeneic models of transplantable murine ovarian adenocarcinoma: ID8, Ig10, M0505, and STOSE [32–34]. STOSE cells are a spontaneously transformed derivative of the immortalized but non-tumorigenic M0505 cell line (courtesy of Dr. Barbara Vanderhyden, Ottowa Hospital Research Institute) [35], which share many molecular properties with human epithelial ovarian carcinoma. Established paired chemo-sensitive, A2780ip2, HEYA8, and SKOV3ip1, and chemo-resistant, A2780cp20, HeyA8MDR, and SKOV3TRip2, (courtesy of Dr. Charles Landen, University of Virginia) human ovarian adenocarcinoma cell lines were treated with oHSV [36, 37]. Individual cell lines were incubated in 225 mL sterile flasks in RPMI plus 10 % FBS as liquid medium (Hyclone, Logan, UT).

### Assessment of in vitro oHSV cytotoxic effect

To assay the cytotoxic effects of oHSV, ovarian cancer cells were harvested from tissue culture after trypsinization, re-suspended in equal volume culture medium and spun down using a centrifuge at (180 × G, 6 min, ambient). The excess liquid was aspirated and the pellet was re-suspended in tissue culture, and a 0.04 % trypan blue exclusion count was performed to calculate the number of viable (dye-excluding) cells per mL. The cell concentration was adjusted so that 4,000 cells were plated in wells A2-H12 of a 96 well plate. The cells were incubated overnight and a 7 tube dilutional series for each virus was performed, evaluating 100, 33, 10, 3.3, 1.0, 0.33, and 0.1 plaque forming units per cell (pfu/well). Two replicate 96 well plates were tested for each virus. The control well, A1, received 200 μL of culture medium only, to “blank” the spectrophotometer and to represent 100 % cytotoxicity, while wells A2-A12 received 100 μL of medium to represent the 100 % (maximum) cell survival group as no virus was added to each this row. Beginning with the most dilute virus (0.01 pfu/cell), 100 μL of virus was added to each well of row B of the plate and continued down the plate for each successive virus concentration. Plates were reincubated for 48 h at which point 25 μL of sterile Alamar Blue dye was added to each well and the BioTek spectrophotometer was used to determine the corrected optical density (OD_562_–OD_590_) of each well 4 and 8 hours after the dye was added. Mean OD values for each virus dilution was used to construct a dose response plot and the number of plaque forming units of virus needed to kill 50 % of the cells (PFU/TD_50_) was calculated utilizing a regression analysis for the linear portion of the sigmoidal plots. Triplicate wells for each virus were obtained and the PFU/TD_50_ was averaged for all three experiments.

### Mouse husbandry and genotyping

MISIIR-TAg mice [38] were obtained from Dr. Denise Connolly, Fox Chase Cancer Center. Transgene-positive male mice were bred to non-transgenic C57BL/6 females and the offspring were genotyped by PCR using DNA extracted from ear punches. DNA was subjected to PCR using the primers, TGCATGGTGTACAACATTCC and TTGGGACTGTGAATCAATGCC, and Expand High Fidelity taq polymerase (Roche) with 30 cycles of 95 °C for 30s, 60 °C for 30s and 72 °C for 30s. The resulting amplicon was analyzed by agarose gel electrophoresis and a band of 773 bp was indicative of a positive reaction. Only transgene positive females were used in our study since these mice are known to develop spontaneous ovarian cancer with metastases to the omentum as early as 7 weeks [39]. All experiments involving mice were approved by the University of Alabama at Birmingham Institutional Animal Care and Use Committee.

### Assessment of anti-tumor effect in a spontaneous ovarian cancer in vivo model

Transgene positive female MISIIR-TAg mice (*n* = 22) were randomized into control and experimental groups at 3 weeks of age and underwent bilateral salpingo-oophorectomy at 10 weeks. 1, 2, and 3 weeks after surgery, mice received either 1 × 10^7^ PFU of M002 oHSV or 500 ul phosphate buffer solution (PBS) in the peritoneal cavity. The oHSV dose (1 × 10^7^ PFU) used in this study was noted in previous experiments to be the maximum tolerated dose in the HSV-sensitive A/J mouse strain [12]. Mice were monitored using standard American Association for Laboratory Animal Science (IACUC) body condition scoring until they reached the study endpoint of 6 months; remaining mice were euthanized and metastatic disease, if present, was measured. Mice with a body condition score of less than 2 were euthanized and counted as “dead” if there was evidence of tumor at the time of necropsy. Overall survival (OS) was defined as time from bilateral salpingo-oophorectomy to death from any cause.

### Assessment of immune response in a spontaneous ovarian cancer in vivo model

In a separate assessment we randomized 36 additional MISIIR-TAg mice to receive either 1 × 10^7^ PFU of M002 oHSV or 500 ul PBS IP and quantified the number of tumor antigen-specific CD8^+^ T-cells in the omentum, peritoneal cavity, lymph nodes, and spleen at 7 or 10 days. Specifically, cell suspensions were obtained for these studies by mechanical disruption from mediastinal lymph nodes and spleen, and by enzymatic digestion of the omentum (5 mg/mL collagenase-DNase I mix (Sigma Aldrich®, Inc. St. Louis, MO) in DMEM media at 37 °C). Cells from the peritoneal cavity for these studies were collected from ice-cold PBS washings obtained from the peritoneal cavity. All cell suspensions were blocked with Fc block for 10 min at 4 °C, followed by incubation with anti-CD8 (clone, Biolegend), anti-CD3 (clone, eBioscience), and the MHC class-I tetramers, H2K^b^-(SV40Tag LIWIPALL) and H2K^b^-(Mesothelin AGINNLDNL), both from the NIH tetramer core facility. Flow cytometry was performed using a FACSCanto II (BD Bioscience) and data were analysed using FlowJo software (Tree Star, Inc.).

### Statistical analysis

The difference between group proportions was based on the Fisher exact test or unpaired student’s *t*-test with equal SD. Survival curves were based on Kaplan-Meier estimates. All statistical tests were two-sided and a 5 % significance level was assumed for all tests. Graphs and statistical analysis were performed using Prism software version 6.0 (Graphpad Software, Inc. La Jolla, CA).

## Results

### oHSVs demonstrate broad cytotoxicity against in vitro models of ovarian cancer

**Fig. 1:**
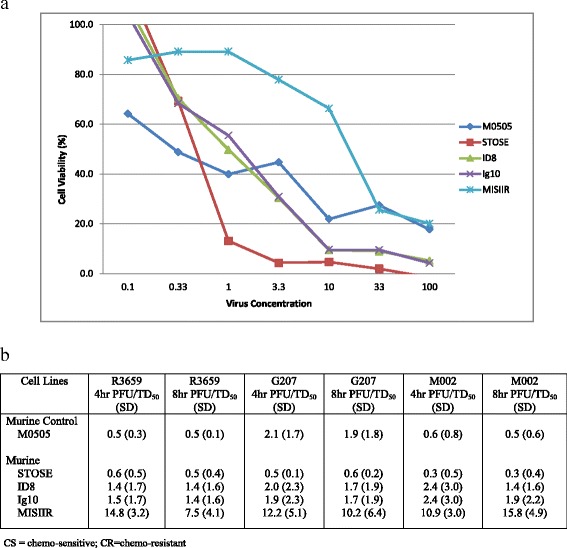
In vitro 4 h cytotoxicity of oHSV towards murine ovarian cancer cell lines. 48 h after M002 was added to the incubating murine cell lines, Alamar Blue dye was added to each well. 4 and 8 h after the Alamar Blue dye was added to the wells, the mean optical density for each well was calculated and used to construct a dose response plot and the number of viable cells was calculated utilizing regression analysis. A representative figure at 4 h demonstrates the cytotoxicity of M002 towards the syngeneic murine cell lines (**a**), and the mean number of PFU/TD_50_ at 4 and 8 h is demonstrated (**b**)

**Fig. 2:**
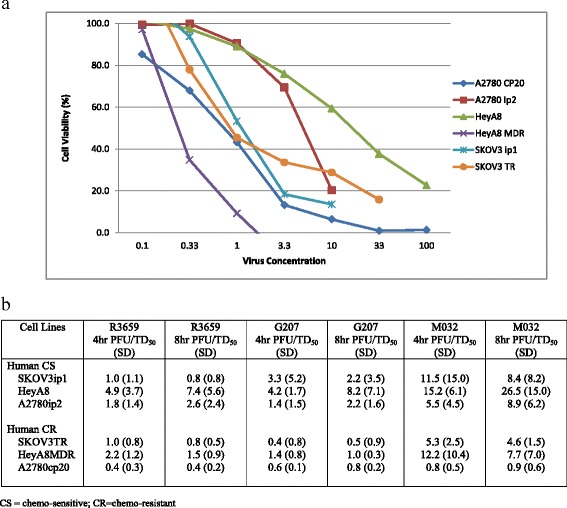
In vitro 4 h cytotoxicity of oHSV towards human ovarian cancer cell lines. 48 h after M032 was added to the incubating murine cell lines, Alamar Blue dye was added to each well. 4 and 8 h after the Alamar Blue dye was added to the wells, the mean optical density for each well was calculated and used to construct a dose response plot and the number of viable cells was calculated utilizing regression analysis. A representative figure at 4 h demonstrates the cytotoxicity of M032 towards the human ovarian cancer cell lines (**a**), and the mean number of PFU/TD_50_ at 4 and 8 h is demonstrated (**b**)

We evaluated the capacity of several oHSV (R3659, G207, M032, and M002) at various dose concentrations to achieve oncolysis in a variety of mouse and human ovarian cancer cell lines. Using data generated by the Alamar Blue assay, we were able to develop linear regression from the dose response plots. The paired human chemo-sensitive (CS) and chemo-resistant (CR) cell lines and MISIIR transgenic mouse cancer cells all demonstrated susceptibility to the anti-tumor cytotoxicity of oHSV in vitro. Certain murine cell lines were more sensitive to the cytotoxic effects of oHSV, with STOSE and ID8 demonstrating extensive cytotoxicity across all evaluated viruses (Fig. 1a). The MISIIR cell line appeared less sensitive to oHSV; however, an anti-tumor effect was still observed at higher virus concentrations. The mean PFU/TD_50_ (averaged for each experiment which was repeated twice) for each virus with the standard deviation is demonstrated for each evaluated murine ovarian cancer cell line (Fig. 1b). Within the human CS lines, SKOV3ip1 and A2780ip2 demonstrated greater cytotoxicity to all oHSVs when compared to HEYA8. All three human CR cell lines were exquisitely sensitive to oHSV, with A2780cp20 and SKOV3TR being the most susceptible to M032 (Fig. 2a). The mean PFU/TD_50_ (averaged for each experiment which was repeated twice) for each virus is demonstrated for the human ovarian cancer cell lineages (Fig. 2b).

### *oHSV demonstrates decreased rates of metastatic ovarian cancer* in in vivo *models*

**Fig. 3:**
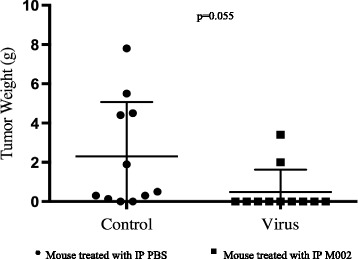
Anti-tumor effect in MISIIR-TAg mice treated with IP M002 or PBS in vivo MISIIR mice were injected with IP M002 or PBS weekly for a total of three doses. They were followed until death at which time a necropsy was performed to determine the presence and extent of tumor burden. The weight of each mouse’s tumor is depicted. Each circle represents a mouse treated with IP PBS, whereas each square represents a mouse treated with IP M002. Two (18 %) mice treated with PBS were without evidence of tumor compared to 9 (81 %) mice treated with M002 (*p* = 0.055)

**Fig. 4:**
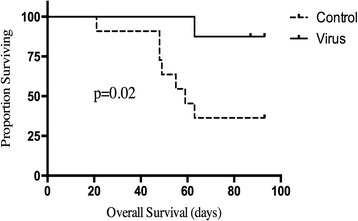
Overall survival in MISIIR-TAg mice treated with IP M002 or PBS. MISIIR mice were observed following 3 weekly treatments with IP M002 or PBS. Necropsy was performed at time of death evaluating for the presence of metastatic ovarian carcinoma. If the mice were still alive at 6 months, a planned euthanasia date to evaluate for metastatic disease was predetermined prior to mouse randomization. Mice treated with M002 had a significantly improved overall survival compared to those treated with placebo (*p* = 0.02)

To assess the ability of oHSV to modulate overall survival and tumor response in a spontaneous model of ovarian carcinoma, MISIIR mice were randomized to receive 3 weekly IP injections of M002 or PBS. Mice were observed until they exceeded their clinical endpoint (counted as death) or were euthanized at the designated 6 month study end. In the experimental cohort (M002 treated) only 2 mice developed metastatic disease; one was euthanized on post-operative day 63 and was found to have tumor weighing 2.0 g, the other was euthanized at the 6 month endpoint and had occult 3.39 g of tumor without ascites. Five (45.5 %) mice treated with M002 were euthanized at 6 months and did not have any evidence of metastatic tumor. Within the control group (PBS), two of the mice were euthanized at 6 months and were found to have ascites and tumors weighing 1.9 g and 0.53 gm, respectively. The remaining seven control mice were euthanized with metastatic disease and had tumor weights ranging from 0.14 to 7.8 gm (Fig. 3). Compared to the control group, mice treated with M002 were less likely to develop metastatic ovarian cancer (81.2 % vs 18.2 %; *p* = 0.008) and had a significantly longer OS (*p* = 0.02; Fig. 4). Seven (77.8 %) control mice had ascites at the time of necropsy compared to one (9 %) mouse treated with oHSV. One mouse treated with M002 died on post-operative day 21 of unknown causes; however, there was no evidence of tumor at necropsy. Three (27.3 %) mice treated with M002 developed torticollis and were ultimately euthanized on post-operative days 27, 37, and 60 secondary to declining body condition scoring. This was in comparison to 1 control mouse euthanized on post-operative day 55, secondary to declining body condition score, which exhibited metastatic disease at time of necropsy.

### oHSV demonstrates robust tumor antigen-specific CD8^+^ T-cell immune response within the peritoneal cavity

To evaluate the tumor antigen-specific CD8^+^ T-cell immune response, additional MISIIR mice were randomized to receive 3 weekly IP injections of the IL-12 expressing oHSV, M002, or PBS. 7 or 10 days following the 3rd injection, the mice were euthanized and the number of tumor antigen-specific CD8^+^ T-cells in the omentum, peritoneal cavity, lymph nodes, and spleen were quantified. When compared to the control mice, mice injected with M002 demonstrated a more robust tumor antigen-specific CD8^+^ T-cell immune response in most evaluated tissues for both days 7 and 10.

**Fig. 5:**
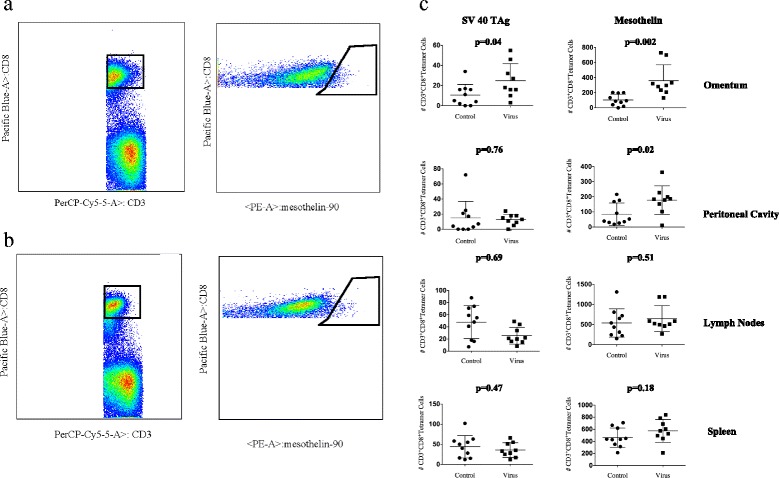
Day 7 tumor antigen-specific CD8^+^ T cell immune response. MISIIR mice were treated with either IP M002 or PBS weekly for a total of 3 weeks. Mice were subsequently euthanized on day 7 or day 10 following the third injection. A representative day 7 flow cytometry gate demonstrating the number of tumor antigen-specific CD8^+^ T-cells for the Mesothelin tetramer in the omentum is illustrated for mice treated with M002 (**a**) and PBS (**b**). The day 7 tumor antigen-specific CD8^+^ T-cell immune response for the SV40-TAg and Mesothelin tetramer from the omentum, peritoneal fluid, draining lymph nodes, and spleen is demonstrated. The number of antigen-specific CD8^+^ T-cells was significantly larger to the SV40-TAg in the omentum (*p* = 0.04) and to Mesothelin in the omentum (*p* = 0.002) and peritoneal cavity (*p* = 0.02) (**c**)

A robust immune response in mice treated with M002 was observed within the day 7 cohort. The day 7 flow cytometry numbers of tumor antigen-specific CD8^+^ T-cells for the SV40-TAg and Mesothelin tetramers in the omentum are illustrated for mice treated with IP M002 (Fig. 5a) and PBS (Fig. 5b). The number of tumor antigen-specific CD8^+^ T-cells was significantly higher for the SV40-TAg in the omentum and to mesothelin in both the omentum and peritoneal cavity. Specifically, the number of tumor antigen-specific CD8^+^ T-cells to the SV40-TAg in the omentum was 24.8 vs 10.5 (*p* = 0.04) and the number of tumor antigen-specific CD8^+^ T-cells to mesothelin in the omentum and peritoneal cavity were 359.1 vs 105.0 (*p* = 0.002) and 178.0 vs 85.3 (*p* = 0.02) respectively. While not significant a more pronounced immune response in mice treated with M002 was observed in both the lymph nodes and spleen towards the Mesothelin tetramer (Fig. 5c).

**Fig. 6:**
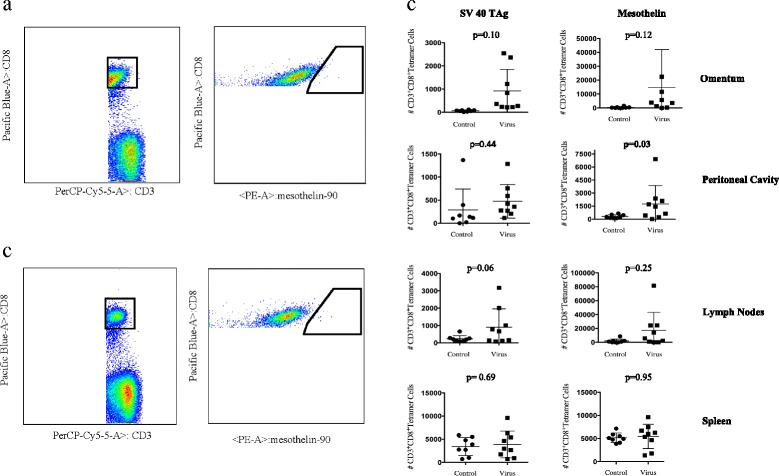
Day 10 tumor antigen-specific CD8^+^ T cell immune response. MISIIR mice were treated with either IP M002 or PBS weekly for a total of 3 weeks. Mice were subsequently euthanized on day 7 or day 10 following the third injection. A representative day 10 flow cytometry gate demonstrating the numbers of tumor antigen-specific CD8^+^ T-cells for the Mesothelin tetramer in the omentum is illustrated for mice treated with M002 (**a**) and PBS (**b**). The day 10 tumor antigen-specific CD8^+^ T-cell immune response for the SV40-TAg and Mesothelin tetramer from the omentum, peritoneal fluid, draining lymph nodes, and spleen is demonstrated. The number of antigen-specific CD8^+^ T-cells was significantly larger to Mesothelin in the peritoneal cavity (*p* = 0.03) (**c**)

Similar to the analysis of the day 7 cohort, M002 treated mice euthanized and analyzed at day 10 demonstrated an increased immune response within the peritoneal cavity compared to mice treated with PBS. The day 10 flow cytometry numbers of tumor antigen-specific CD8^+^ T-cells for the SV40-TAg and mesothelin tetramers in the omentum are illustrated for mice treated with IP M002 (Fig. 6a) and PBS (Fig. 6b). The number of tumor antigen-specific CD8^+^ T-cells to the mesothelin-tetramer in the peritoneal cavity was significantly higher in mice treated with M002 compared to mice treated with PBS, 1009.0 vs 246.0 (*p* = 0.03). Although not significant, the number of tumor antigen-specific CD8^+^ T-cells to the SV40-TAg-tetramer and mesothelin-tetramer in the omentum, peritoneal cavity, and lymph nodes was more potent in mice treated with M002 (Fig. 6c).

## Discussion

Oncolytic viruses are an emerging immunotherapeutic approach for cancer as demonstrated by the recent FDA approval of talimogene laherparepvec (T-VEC) (Amgen, Imlygic®), the first FDA-approved oncolytic virus therapy for the treatment of advanced and unresectable melanoma [23]. Studies utilizing oncolytic reovirus, measles vaccine virus, vaccinia virus, vesicular stomatitis virus, and adenovirus have demonstrated the feasibility of this approach for ovarian cancer [40]. HSV is another reasonable oncolytic to evaluate for the treatment of ovarian cancer. Our translational pilot study provides preliminary evidence supporting that oHSV may be an effective treatment agent for women with ovarian cancer. In our study, all evaluated oHSVs (R3659, G207, M032, and M002) demonstrated a cytotoxic effect in vitro to a variety of mouse and human ovarian cancer cell lines. Although some cell lines demonstrated greater sensitivity to oHSV (STOSE, Ig10, M0505, & A2780cp20), a cytotoxic anti-tumor effect was observed across all lineages. Importantly, each oHSV demonstrated an anti-tumor response towards the three human CR cell lines. Since our cytotoxicity studies were performed in vitro*,* it is not surprising that the “armed” oHSV did not outperform the “unarmed” viruses. In our preliminary in vivo studies, IP injection of M002 was associated with a significant improvement in OS when compared to IP injection with PBS. A significant difference in the number of mice that died with metastatic ovarian cancer was observed between the two cohorts. Furthermore, treatment with 3 weekly doses of M002 led to less accumulation of ascites. IP injection with M002 was reasonably tolerated. Although three mice in the experimental cohort developed torticollis, this was not significantly different from the control group. The basis for this head and neck dystonia was not readily apparent, and we recognize that it is much higher than the observed rate of 2 % of transgene positive MISIIR mice within our breeding colony. We cannot state that either the virus or the IL-12 could have caused this change. Although we did not examine the inner ear, the brain or brain stem in these mice, we would speculate that non-infected metastatic ovarian tumor cells, escaping from the peritoneum, might have been the cause. In future studies, complete necropsies of mice exhibiting head tilt will be conducted to establish the basis. The dose used in this study was based upon previous work that demonstrated maximum LD_50_ without increased toxicity [12]. It is yet to be determined if smaller doses of virus would be as efficacious.

The results of our studies are similar to that of others. Specifically, preclinical studies have demonstrated that IP injection of HSV-1716-infected PA-1 carrier cells led to a significant tumor reduction in SCID mice that had been inoculated with A2780 cells and a significant prolongation of median survival in SCID mice bearing SKOV3 tumors (17 weeks vs 9 weeks) [21]. Utilizing a murine syngeneic model of ovarian cancer, Benencia et al. demonstrated that intratumoral injection of HSV-1716 resulted in reduction of tumor growth and a significant survival advantage [41]. Additionally, Coukos and colleagues demonstrated that IP injection of HSV-G207, a recombinant HSV-1 lacking the infected cell products 34.5 and 6, which enhances tumor cell selectivity, was associated with a significant reduction in tumor volume and tumor spread in an in vivo mouse xenograft model of ovarian cancer [22]. To further the therapeutic potential of replication-selective viruses, Fu et al. constructed an oncolytic virus from HSV-2 by deleting the protein kinase domain of the viral ICP10 gene, which targets the Ras signaling pathway in tumor cells. HSV-2 was injected IP into nude mice bearing metastatic human ovarian tumor xenografts and demonstrated complete tumor abolition in 87 % of the mice [42]. Wang and colleagues demonstrated that HSV-1 thymidine kinase mediated gene transfer was superior to adenoviral vectors and required lower does in vitro, supporting HSV-1 as a capable and alternative vector for ovarian cancer gene therapy [43]. There is currently a phase I trial (NCT01997190) evaluating AdV-tk (Advantagene, Inc), an oncolytic herpes simplex virus in patients with malignant pleural effusions and solid tumors, including ovarian cancer. The optimal use of adjuvant IL-12 in women with advanced ovarian cancer has not been determined. For example, Lenzi el al. evaluated IP rIL-12 in women with advanced ovarian cancer who completed platinum-based chemotherapy and underwent a second look surgery. Women with ≤ 1 cm residual disease were treated with IP rIL-12 at weekly intervals with a treatment range of only 1 to 16 weeks. Of 11 women that were assessed, 2 had stable disease while 9 had progressive disease. Although the response rate was worse than anticipated, this patient population did significantly worse than what is traditionally observed in women with optimally debulked ovarian cancer [44]. Future studies exploring the optimal patient population with advanced ovarian cancer, tumor biology/histology, pleiotrophic effects of IL-12, and combining IL-12 with oncolytic virus are warranted.

The omentum has several immunogenic roles xmediated primarily by filtering peritoneal fluid, pathogens, antigens, and cells, including metastasizing ovarian cancer cells [45, 46]. The omentum contains a variety of immune cells (T cells, B cells, and dendritic cells) that are clustered in milky spots embedded between adipocytes. These milky spots detect peritoneal antigens and generate immune responses but also make them an ideal site for collecting metastases from peritoneal cancers. Metastatic tumor cells have been demonstrated in the omentum in MISIIR mice as early as 7 weeks. One might expect that if tumor cells are trapped in milky spots and milky spots are immune inductive sites, then the omentum should trigger an anti-tumor immune response. However, Tregs also accumulate in the omentum of mice together with metastatic carcinoma, suggesting that the omentum may be a site of immune suppression rather than immune activation. Additionally, specialized populations of Tregs reside in visceral adipose tissue, including the omentum. These Tregs are dependent on the transcription factor PPARg and are profoundly suppressive. In addition, innate lymphocyte cells (ILCs) that make IL-13 also reside in the omentum. Both the ILCs and the Tregs are likely to suppress anti-tumor immunity [47–52]. Via this filtering process, milky spots within the omentum are ideal sites for peritoneal cancer cells to collect and influence the host immune response [15, 16, 53]. The microenvironments of the omentum and peritoneal cavity are unique milieu’s that can potentially be altered for therapeutic gains. Agents that modify or reverse this process may lead to improved outcomes in women with ovarian cancer. Our results demonstrate that IP treatment with the murine IL-12 expressing oHSV, M002, was associated with a more robust local immune response as demonstrated by higher numbers of tumor antigen-specific CD8^+^ T-cells within the omentum and peritoneal cavity. Compared to mice treated with PBS, mice treated with M002 demonstrated an increased immune response at both 7 and 10 days following their last of 3 weekly injections. Treatment with M002 was also associated with increased tumor antigen-specific CD8^+^ T-cells within the spleen and draining lymph nodes, however it was not as significant, suggesting that the omentum and peritoneal cavity were the primary immune effector sites of the oHSV regardless of timing from last IP injection.

In summary, our data demonstrate that “unarmed” and IL-12 “armed” oHSV produce a vigorous cytotoxic effect across a host of ovarian cancer cell lines in vitro*.* Furthermore, MISIIR mice treated with IP injections of M002 had significantly lower rates of metastatic ovarian cancer and improved survival. Finally, a more robust tumor antigen-specific CD8^+^ T-cell immune response was demonstrated in most tissues treated with oHSV compared to placebo, in spite of limited data defining the optimal M002 dose or schedule. We recognize that additional more in depth studies are required to further clarify the benefit of using an IL-12 expressing oHSV over an unarmed oHSV and to more comprehensively assess the immune response. Additionally, studies evaluating the optimal dosing strategy, characterizing at the molecular level the genetic determinants of sensitivity to oHSV, and assessing novel immunotherapy combination approaches with oHSV appear to be warranted. Nevertheless, this preliminary study demonstrates the potential of an IL-12 expressing oHSV in this disease context.

## Conclusion

Our translational pilot study demonstrates that an IL-12-expressing oHSV effectively kills both murine and human ovarian cancer cell lines in vitro and it promotes tumor antigen-specific CD8^+^ T-cell responses in the peritoneal cavity and omentum. IP injection with M002 appears to be associated with reduced peritoneal metastasis and improved survival in a murine ovarian cancer model. Epithelial ovarian carcinoma continues to be the most lethal gynecologic malignancy in the United States. Despite enhancement in conventional surgical debulking and chemotherapy, most women with advanced disease eventually recur and ultimately die from metastatic disease. To date there are no FDA approved oncolytic viruses for the treatment of ovarian cancer. Consequently, developing novel treatments, such as oncolytic viruses, for women with epithelial ovarian cancer are of upmost importance for women with this disease.

## Abbreviations

CR: Chemoresistant
CS: Chemosensitive
FDA: United States food and drug administration
HSV: Herpes simplex virus
IACUC: American association for laboratory animal science
IL: Interleukin
IP: Intraperitoneal
M002: Murine interleukin-12 expressing oncolytic herpes simplex virus
M032: Human interleukin-12 expressing oncolytic herpes simplex virus
OD: Optical density
oHSV: Oncolytic herpes simplex virus
OS: Overall survival
PBS: Sterile phosphate buffer solution
Pfu: Plaque forming unit
PFU/TD_50_: Plaque forming units of virus needed to kill 50 % of the cells
TIL: Tumor infiltrating lymphocytes
Treg: Regulatory T-cells

## References

[1] Siegel RL, Miller KD, Jemal A (2015). Cancer statistics, 2015. CA Cancer J Clin.

[2] Markman M, Markman J, Webster K, Zanotti K, Kulp B, Peterson G (2004). Duration of response to second-line, platinum-based chemotherapy for ovarian cancer: implications for patient management and clinical trial design. J Clin Oncol Off J Am Soc Clin Oncol.

[3] Sabbatini P, Odunsi K (2007). Immunologic approaches to ovarian cancer treatment. J Clin Oncol Off J Am Soc Clin Oncol.

[4] June CH, Maus MV, Plesa G, Johnson LA, Zhao Y, Levine BL (2014). Engineered T cells for cancer therapy. Cancer Immunol Immunother.

[5] Couzin-Frankel J (2013). Breakthrough of the year 2013. Cancer Immunother Sci.

[6] Hwang WT, Adams SF, Tahirovic E, Hagemann IS, Coukos G (2012). Prognostic significance of tumor-infiltrating T cells in ovarian cancer: a meta-analysis. Gynecol Oncol.

[7] Sato E, Olson SH, Ahn J, Bundy B, Nishikawa H, Qian F (2005). Intraepithelial CD8+ tumor-infiltrating lymphocytes and a high CD8+/regulatory T cell ratio are associated with favorable prognosis in ovarian cancer. Proc Natl Acad Sci U S A.

[8] Hamanishi J, Mandai M, Iwasaki M, Okazaki T, Tanaka Y, Yamaguchi K (2007). Programmed cell death 1 ligand 1 and tumor-infiltrating CD8+ T lymphocytes are prognostic factors of human ovarian cancer. Proc Natl Acad Sci U S A.

[9] Mariya T, Hirohashi Y, Torigoe T, Asano T, Kuroda T, Yasuda K (2014). Prognostic impact of human leukocyte antigen class I expression and association of platinum resistance with immunologic profiles in epithelial ovarian cancer. Cancer Immunol Res.

[10] Paroli M, Bellati F, Videtta M, Focaccetti C, Mancone C, Donato T (2014). Discovery of chemotherapy-associated ovarian cancer antigens by interrogating memory T cells. Int J Cancer.

[11] Curiel TJ, Coukos G, Zou L, Alvarez X, Cheng P, Mottram P (2004). Specific recruitment of regulatory T cells in ovarian carcinoma fosters immune privilege and predicts reduced survival. Nat Med.

[12] Parker JN, Gillespie GY, Love CE, Randall S, Whitley RJ, Markert JM (2000). Engineered herpes simplex virus expressing IL-12 in the treatment of experimental murine brain tumors. Proc Natl Acad Sci U S A.

[13] Hellums EK, Markert JM, Parker JN, He B, Perbal B, Roizman B (2005). Increased efficacy of an interleukin-12-secreting herpes simplex virus in a syngeneic intracranial murine glioma model. Neuro-Oncology.

[14] Portielje JE, Kruit WH, Eerenberg AJ, Schuler M, Sparreboom A, Lamers CH (2005). Subcutaneous injection of interleukin 12 induces systemic inflammatory responses in humans: implications for the use of IL-12 as vaccine adjuvant. Cancer Immunol Immunother.

[15] Krist LF, Kerremans M, Broekhuis-Fluitsma DM, Eestermans IL, Meyer S, Beelen RH (1998). Milky spots in the greater omentum are predominant sites of local tumour cell proliferation and accumulation in the peritoneal cavity. Cancer Immunol Immunother.

[16] Williams R, White H (1986). The greater omentum: its applicability to cancer surgery and cancer therapy. Curr Probl Surg.

[17] Beelen RH, Fluitsma DM, Hoefsmit EC (1980). The cellular composition of omentum milky spots and the ultrastructure of milky spot macrophages and reticulum cells. J Reticuloendothel Soc.

[18] Trinchieri G, Scott P (1995). Interleukin-12: a proinflammatory cytokine with immunoregulatory functions. Res Immunol.

[19] Kim KH, Dmitriev IP, Saddekni S, Kashentseva EA, Harris RD, Aurigemma R (2013). A phase I clinical trial of Ad5/3-Delta24, a novel serotype-chimeric, infectivity-enhanced, conditionally-replicative adenovirus (CRAd), in patients with recurrent ovarian cancer. Gynecol Oncol.

[20] Kimball KJ, Preuss MA, Barnes MN, Wang M, Siegal GP, Wan W (2010). A phase I study of a tropism-modified conditionally replicative adenovirus for recurrent malignant gynecologic diseases. Clin Cancer Res.

[21] Coukos G, Makrigiannakis A, Kang EH, Caparelli D, Benjamin I, Kaiser LR (1999). Use of carrier cells to deliver a replication-selective herpes simplex virus-1 mutant for the intraperitoneal therapy of epithelial ovarian cancer. Clin Cancer Res.

[22] Coukos G, Makrigiannakis A, Montas S, Kaiser LR, Toyozumi T, Benjamin I (2000). Multi-attenuated herpes simplex virus-1 mutant G207 exerts cytotoxicity against epithelial ovarian cancer but not normal mesothelium and is suitable for intraperitoneal oncolytic therapy. Cancer Gene Ther.

[23] Andtbacka RH, Kaufman HL, Collichio F, Amatruda T, Senzer N, Chesney J (2015). Talimogene Laherparepvec Improves Durable Response Rate in Patients with Advanced Melanoma. J Clin Oncol Off J Am Soc Clin Oncol.

[24] Markert JM, Cody JJ, Parker JN, Coleman JM, Price KH, Kern ER (2012). Preclinical evaluation of a genetically engineered herpes simplex virus expressing interleukin-12. J Virol.

[25] Martuza RL, Malick A, Markert JM, Ruffner KL, Coen DM (1991). Experimental therapy of human glioma by means of a genetically engineered virus mutant. Science.

[26] Kramm CM, Chase M, Herrlinger U, Jacobs A, Pechan PA, Rainov NG (1997). Therapeutic efficiency and safety of a second-generation replication-conditional HSV1 vector for brain tumor gene therapy. Hum Gene Ther.

[27] Pyles RB, Warnick RE, Chalk CL, Szanti BE, Parysek LM (1997). A novel multiply-mutated HSV-1 strain for the treatment of human brain tumors. Hum Gene Ther.

[28] Chambers R, Gillespie GY, Soroceanu L, Andreansky S, Chatterjee S, Chou J (1995). Comparison of genetically engineered herpes simplex viruses for the treatment of brain tumors in a scid mouse model of human malignant glioma. Proc Natl Acad Sci U S A.

[29] Markert JM, Malick A, Coen DM, Martuza RL (1993). Reduction and elimination of encephalitis in an experimental glioma therapy model with attenuated herpes simplex mutants that retain susceptibility to acyclovir. Neurosurgery.

[30] Chou J, Roizman B (1990). The herpes simplex virus 1 gene for ICP34.5, which maps in inverted repeats, is conserved in several limited-passage isolates but not in strain 17syn+. J Virol.

[31] Roth JC, Cassady KA, Cody JJ, Parker JN, Price KH, Coleman JM, et al. Evaluation of the Safety and Biodistribution of M032, an Attenuated HSV-1 Virus Expressing hIL-12, After Intracerebral Administration to Aotus Non-Human Primates. Hum Gene Ther Clin Dev. 2014. 10.1089/humc.2013.201 PMC4047998 24649838

[32] Lv L, Zhang T, Yi Q, Huang Y, Wang Z, Hou H (2012). Tetraploid cells from cytokinesis failure induce aneuploidy and spontaneous transformation of mouse ovarian surface epithelial cells. Cell Cycle.

[33] Roby KF, Taylor CC, Sweetwood JP, Cheng Y, Pace JL, Tawfik O (2000). Development of a syngeneic mouse model for events related to ovarian cancer. Carcinogenesis.

[34] Roberts PC, Mottillo EP, Baxa AC, Heng HH, Doyon-Reale N, Gregoire L (2005). Sequential molecular and cellular events during neoplastic progression: a mouse syngeneic ovarian cancer model. Neoplasia.

[35] McCloskey CW, Goldberg RL, Carter LE, Gamwell LF, Al-Hujaily EM, Collins O (2014). A new spontaneously transformed syngeneic model of high-grade serous ovarian cancer with a tumor-initiating cell population. Front Oncol.

[36] Landen CN, Chavez-Reyes A, Bucana C, Schmandt R, Deavers MT, Lopez-Berestein G (2005). Therapeutic EphA2 gene targeting in vivo using neutral liposomal small interfering RNA delivery. Cancer Res.

[37] Thaker PH, Yazici S, Nilsson MB, Yokoi K, Tsan RZ, He J (2005). Antivascular therapy for orthotopic human ovarian carcinoma through blockade of the vascular endothelial growth factor and epidermal growth factor receptors. Clin Cancer Res.

[38] Quinn BA, Xiao F, Bickel L, Martin L, Hua X, Klein-Szanto A (2010). Development of a syngeneic mouse model of epithelial ovarian cancer. J Ovarian Res.

[39] Connolly DC, Bao R, Nikitin AY, Stephens KC, Poole TW, Hua X (2003). Female mice chimeric for expression of the simian virus 40 TAg under control of the MISIIR promoter develop epithelial ovarian cancer. Cancer Res.

[40] Hartkopf AD, Fehm T, Wallwiener D, Lauer U (2011). Oncolytic virotherapy of gynecologic malignancies. Gynecol Oncol.

[41] Benencia F, Courreges MC, Conejo-Garcia JR, Mohamed-Hadley A, Zhang L, Buckanovich RJ (2005). HSV oncolytic therapy upregulates interferon-inducible chemokines and recruits immune effector cells in ovarian cancer. Mol Ther.

[42] Fu X, Tao L, Zhang X (2007). An oncolytic virus derived from type 2 herpes simplex virus has potent therapeutic effect against metastatic ovarian cancer. Cancer Gene Ther.

[43] Wang M, Rancourt C, Navarro JG, Krisky D, Marconi P, Oligino T (1998). High-efficacy thymidine kinase gene transfer to ovarian cancer cell lines mediated by herpes simplex virus type 1 vector. Gynecol Oncol.

[44] Lenzi R, Edwards R, June C, Seiden MV, Garcia ME, Rosenblum M (2007). Phase II study of intraperitoneal recombinant interleukin-12 (rhIL-12) in patients with peritoneal carcinomatosis (residual disease < 1 cm) associated with ovarian cancer or primary peritoneal carcinoma. J Transl Med.

[45] Sorensen EW, Gerber SA, Sedlacek AL, Rybalko VY, Chan WM, Lord EM (2009). Omental immune aggregates and tumor metastasis within the peritoneal cavity. Immunol Res.

[46] Fedorko ME, Hirsch JG (1971). Studies on transport of macromolecules and small particles across mesothelial cells of the mouse omentum. I. Morphologic aspects. Exp Cell Res.

[47] Kolodin D, Van Panhuys N, Li C, Magnuson AM, Cipolletta D, Miller CM (2015). Antigen-and cytokine-driven accumulation of regulatory T cells in visceral adipose tissue of lean mice. Cell Metab.

[48] Cipolletta D (2014). Adipose tissue-resident regulatory T cells: phenotypic specialization, functions and therapeutic potential. Immunology.

[49] Cipolletta D, Cohen P, Spiegelman BM, Benoist C, Mathis D (2015). Appearance and disappearance of the mRNA signature characteristic of Treg cells in visceral adipose tissue: age, diet, and PPARgamma effects. Proc Natl Acad Sci U S A.

[50] Cipolletta D, Kolodin D, Benoist C, Mathis D (2011). Tissular T (regs): a unique population of adipose-tissue-resident Foxp3 + CD4+ T cells that impacts organismal metabolism. Semin Immunol.

[51] Burzyn D, Benoist C, Mathis D (2013). Regulatory T cells in nonlymphoid tissues. Nat Immunol.

[52] Molofsky AB, Van Gool F, Liang HE, Van Dyken SJ, Nussbaum JC, Lee J (2015). Interleukin-33 and Interferon-gamma Counter-Regulate Group 2 Innate Lymphoid Cell Activation during Immune Perturbation. Immunity.

[53] Rangel-Moreno J, Moyron-Quiroz JE, Carragher DM, Kusser K, Hartson L, Moquin A (2009). Omental milky spots develop in the absence of lymphoid tissue-inducer cells and support B and T cell responses to peritoneal antigens. Immunity.

